# Supercooling of Water Controlled by Nanoparticles and Ultrasound

**DOI:** 10.1186/s11671-018-2560-z

**Published:** 2018-05-10

**Authors:** Wei Cui, Lisi Jia, Ying Chen, Yi’ang Li, Jun Li, Songping Mo

**Affiliations:** 1 School of Materials and Energy, Guangdong University of Technology, No. 100 Waihuan Xi Road, Guangzhou Higher Education Mega Center, Panyu District, Guangzhou, 510006 China; 2 College of Power Engineering, Chongqing University, No. 174 Shazheng Street, Shapingba District, Chongqing, 400044 China

**Keywords:** Water, Solidification, Supercooling, Nanoparticles, Ultrasound

## Abstract

Nanoparticles, including Al_2_O_3_ and SiO_2_, and ultrasound were adopted to improve the solidification properties of water. The effects of nanoparticle concentration, contact angle, and ultrasonic intensity on the supercooling degree of water were investigated, as well as the dispersion stability of nanoparticles in water during solidification. Experimental results show that the supercooling degree of water is reduced under the combined effect of ultrasound and nanoparticles. Consequently, the reduction of supercooling degree increases with the increase of ultrasonic intensity and nanoparticle concentration and decrease of contact angle of nanoparticles. Moreover, the reduction of supercooling degree caused by ultrasound and nanoparticles together do not exceed the sum of the supercooling degree reductions caused by ultrasound and nanoparticles separately; the reduction is even smaller than that caused by ultrasound individually under certain conditions of controlled nanoparticle concentration and contact angle and ultrasonic intensity. The dispersion stability of nanoparticles during solidification can be maintained only when the nanoparticles and ultrasound together show a superior effect on reducing the supercooling degree of water to the single operation of ultrasound. Otherwise, the aggregation of nanoparticles appears in water solidification, which results in failure. The relationships among the meaningful nanoparticle concentration, contact angle, and ultrasonic intensity, at which the requirements of low supercooling and high stability could be satisfied, were obtained. The control mechanisms for these phenomena were analyzed.

## Introduction

The quest for new technologies to avert the increasing concern on environmental problems, the imminent energy shortage, and the high cost of energy and new power plants has been a scientific concern over the last three decades. The main challenge is the lack of storage for excess energy to prevent it from being disposed and to bridge the gap between energy generation and consumption. Latent heat thermal energy storage is a particularly interesting technique because it provides high energy storage density [1]. Water is one of the most common materials used for latent heat storage in practice. It has a high volumetric thermal storage density due to its high latent heat and thermal conductivity. However, one of the major disadvantages of water, as reported by many researchers, has been the supercooling that occurs during solidification processes. Supercooling leads to reduced cooling temperatures; thus, the latent heat will be released at lower temperatures. As a result, large temperature difference between charging and discharging is needed to utilize fully the latent heat, which is undesirable for efficient thermal energy storage applications [2]. Thus, finding methods to reduce the supercooling degree of water is fundamental to advance latent heat thermal energy storage technology.

In the last decade, using nanoparticles as nucleating agents is the widespread and leading method that researchers have adopted to control the supercooling degrees of water. The commonly used nanoparticles are metal and metal oxide, such as TiO_2_, Al_2_O_3_, Cu, and CuO [3–6]. These nanoparticles are hydrophilic and can facilitate the formation of ice nuclei by decreasing the Gibbs free energy of nucleation. Other hydrophobic nanoparticles, such as carbon nanotubes and graphene nanoplatelets, have also been used as nucleating agents by some researchers [7–9]. The reduction of supercooling degree of water is attributed to the high specific surface areas of nanoparticles, which can provide more nucleation sites and increase the nucleation probability at high temperatures. According to the literature, different nanoparticles have different nucleation effects; moreover, nanoparticles with high specific areas can eliminate the supercooling of water, whereas those nanoparticles with hydrophilicity not. For example, adding a small amount of graphene nanoplatelets (0.02 wt.%) can eliminate the supercooling of water [8], whereas a reduction of only 70.9% in supercooling degree can be achieved by using TiO_2_ nanoparticles (1.0 wt.%) [4]. Thus, increasing the number of foreign nucleation sites can be a better method to control the supercooling of water, compared with improving the hydrophilicity of nucleating agents.

Using nanoparticles with high specific surface areas and increasing the concentration of hydrophilic nanoparticles are two common ways of increasing the nucleation sites for water solidification. However, maintaining the dispersion of nanoparticles with high specific area in water is extremely difficult, and nanoparticles tend to aggregate together spontaneously to reduce the surface free energy [10]. Poor dispersion stability of nanoparticles with high specific areas will cause some severe problems in their applications, such as degradation in thermal properties in long-term thermal cycling. The aggregation phenomenon cannot also be avoided when the nanoparticle concentration increases to some extent [11]. For the metal and metal oxide nanoparticles, the estimated critical concentration is approximately 1.0–2.0 wt.%. Therefore, finding other ways to increase the effective sites for water nucleation is necessary.

Applying ultrasound in the solidification has been proven to be an effective method to reduce the supercooling degree of water over the past few years [12]. Ultrasound, when passing through a liquid medium, causes mechanical vibration of liquid. If the liquid medium contains dissolved gas nuclei, which will be the case under normal conditions, the liquid medium can be grown and collapsed by the action of the ultrasound. The phenomenon of growth and collapse of microbubbles under an ultrasonic field is known as “acoustic cavitation” [13]. The ice nucleation of water is generally believed to be closely related to acoustic cavitation. Some researchers consider that the pressure change associated with the collapse of cavitation bubbles may be the reason for the nucleating effect of ultrasound [14–20], whereas others believe that the reduced supercooling degree of water may be due to the cavitation bubble surfaces provided, acting as foreign nucleation sites [21–23]. Further investigations are therefore required to better understand the ultrasound-controlled ice nucleation.

Recently, Liu et al. conducted experiments on water solidification influenced by nanoparticles (i.e., graphene oxide) and ultrasound simultaneously [24]. They found that the supercooling degree of water is reduced more significantly under the combined effect of nanoparticles and ultrasound than that caused by either nanoparticles or ultrasound. However, this interesting phenomenon was not explained well in their study and was generally attributed to the cavitation effect of ultrasound. Our previous work has demonstrated that introducing TiO_2_ nanoparticles and ultrasound into the solidification process can reduce the supercooling degree of water. The higher the ultrasonic power is, the lower the supercooling degree is [25]. However, we also found that the aforementioned aggregation problem appears in water solidification assisted by ultrasound and TiO_2_ nanoparticles; that is, nanoparticles and bubbles tend to be pushed away by the advancing ice-water interface and finally cluster together in the middle of the container, especially at high ultrasonic powers. This finding suggests that ultrasonic power should be set carefully in order to achieve the low supercooling degree and good nanoparticle stability simultaneously. To date, few studies have been reported on the solidification of water assisted by nanoparticles and ultrasound. Thus, conducting detailed investigation to identify and elucidate the combined effect of nanoparticles and ultrasound is deemed necessary.

In the present study, Al_2_O_3_ and SiO_2_ nanoparticles, which are hydrophilic and can be steadily dispersed in water, were adopted, and ultrasound was introduced in the solidification processes of the two aqueous suspensions. The effects of nanoparticle concentration and ultrasonic intensity on the supercooling degree of water were investigated. This study mainly aims to identify the roles that the nanoparticles and ultrasound may play in water solidification and determine the proper nucleation method and corresponding control conditions that can meet the requirements of low supercooling degree and good suspension stability simultaneously. The nucleation mechanism concerning cavitation bubbles was also discussed to show the manner in which nanoparticles and ultrasound affect water solidification.

## Experimental

**Fig. 1:**
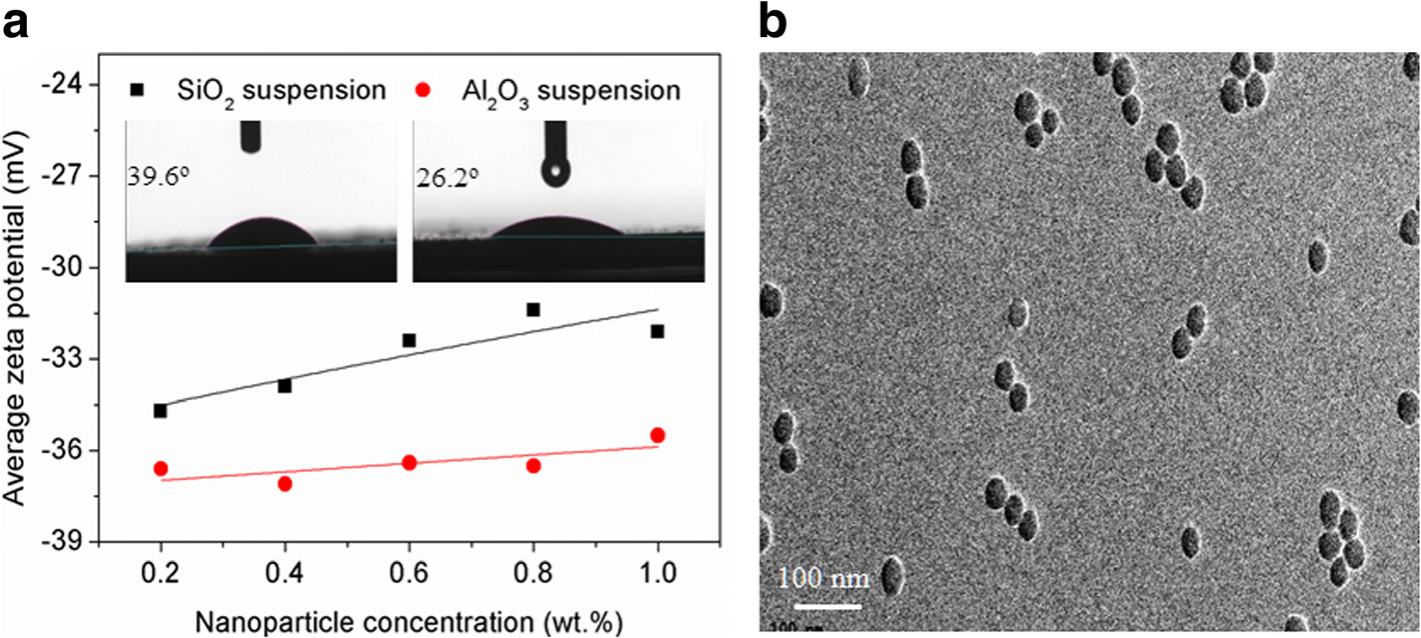
**a** Zeta potentials of the aqueous suspensions of Al_2_O_3_ and SiO_2_ nanoparticles. **b** Typical TEM image of the aqueous suspension of SiO_2_ nanoparticles

Hydrophilic Al_2_O_3_ and SiO_2_ nanoparticles (Aladdin Chemical Reagent Co. Ltd., China) were selected as the nucleating agents in this study, on the basis of their strong affinity to water. The angle of contact between nanoparticles and water was measured using a static sessile drop method with a contact angle goniometry (DataPhysics OCA40 Micro, Germany). Five tests were performed for each nanoparticle, and an average value was obtained from these tests. The contact angle measurements were repeatable within 1% of the mean values, and the measured results are presented in Fig. 1. In preparing nanoparticle suspensions, deionized water was used as the base fluid with the pH adjusted to 8 by sodium hydroxide of analytical grade, and no surfactant was used. An ultrasonication probe (Sonics Vibra Cell, Ningbo Kesheng Ultrasonic Equipment Co. Ltd., China) with 600 W output power and 20 kHz frequency of power supply was applied to disperse the nanoparticles into the deionized water by vibration for 1 h. The nanoparticle concentrations were set to 0.2, 0.4, 0.6, 0.8, and 1.0 wt.%.

A well-dispersed aqueous suspension of nanoparticles can be acquired with a high zeta potential to obtain a strong electrostatic repulsive force. Nanoparticle suspensions with zeta potentials greater than + 30 mV or greater than − 30 mV are normally considered stable in the literature [26]. Thus, the zeta potentials of the aqueous suspensions of Al_2_O_3_ and SiO_2_ nanoparticles at different concentrations were measured using a Zetasizer Nano ZS particle size analyzer (Malvern Instruments Ltd., England). The results are shown in Fig. 1a. The measurements were repeated three times, and the reproducibility of data fell within an error of 1.5%. All the nanoparticle suspensions have a zeta potential higher than − 30 mV, suggesting that the Al_2_O_3_ and SiO_2_ nanoparticles can be steadily dispersed in the water. A transmission electron microscopy (TEM, JEM-100CXII, JEOL, Japan) was further used to measure the nanoparticle distribution in water. Figure 1b shows a typical TEM image of the aqueous suspension of SiO_2_ nanoparticles. Evidently, the nanoparticles are well distributed. In this study, the good dispersion stability of the aqueous suspensions of Al_2_O_3_ and SiO_2_ nanoparticles could be maintained for 4 days without showing any sedimentation signs.

**Fig. 2:**
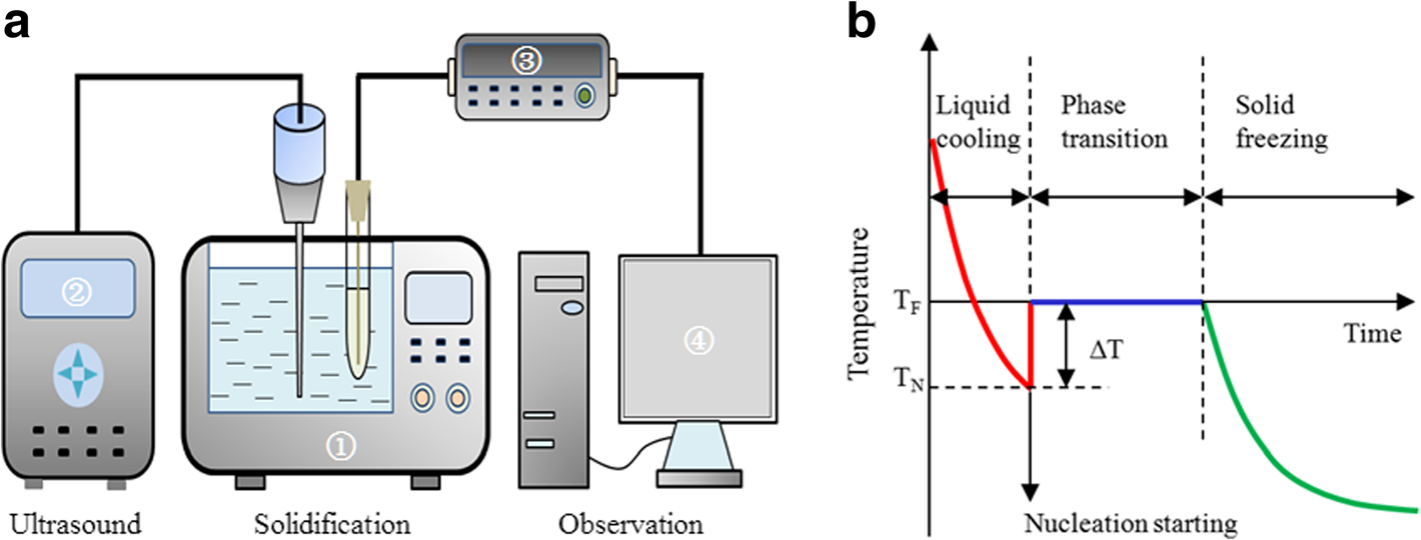
**a** Schematic of the experimental apparatus: (1) thermostatic bath, (2) ultrasonic device, (3) temperature data logger, and (4) computer. **b** Typical temperature profile of water solidification: *T*_F_, freezing temperature; *T*_N_, nucleation temperature; and Δ*T*, supercooling degree (difference between *T*_F_ and *T*_N_)

The experimental apparatus for the water solidification assisted by nanoparticles and ultrasound is schematically shown in Fig. 2a. The following apparatus are as follows: a solidification system consisting of a designed cooling tank and a low-temperature thermostat (CDC-1, Tianjin Huabei Refrigeration Technology Co. Ltd., China) used to freeze samples; an ultrasound-generating system (a commercial ultrasonic device, Sonics Vibra-Cell sonicator JY88-IIN, Ningbo Scientz Biotechnology Co. Ltd., China) used to provide ultrasound fields; an observation system consisting of a temperature data logger (34970A, Agilent Technologies Co. Ltd., USA); and a computer used to monitor the freezing process in real time. To ensure the uniform distribution of ultrasound irradiation, the ultrasonic source was placed vertically in the center of the cooling tank, and the glass container filled with the liquid sample was placed about 2 in. from the ultrasonic source and parallel to it.

In the experiments, the samples of water mixed with and without nanoparticles with a volume of approximately 20 mL were cooled at − 20 °C under different ultrasonic intensities ranging from 0.14 to 1.27 W cm^−2^. The duty cycle of ultrasonic irradiation was set at 80%, representing 8 s on–2 s off. The ultrasound processing began while the sample temperature cooled to 0 °C and finished as soon as ice nucleation occurred in the liquid sample. The ultrasound processing time was very short, less than 2 min. The change in the cooling rate of the liquid sample due to the heat generated by ultrasound was negligible in such a short time. Figure 2b shows a typical temperature profile in solidification. The solidification process can be divided into three subsequent stages, namely, liquid cooling, phase transition, and solid freezing. In the liquid cooling stage, sensible heat is removed from the liquid-state sample and its temperature is lowered. After reaching the freezing point, phase transition is not usually triggered immediately but cooling continues. Therefore, at the end of the precooling stage, the sample remains unfrozen below its freezing point; that is, the sample is supercooled. After a certain degree of supercooling, ice nucleation suddenly occurs. Thereafter, the sample undergoes the phase transition. In this study, a copper–constantan T-type thermocouple with accuracy of ± 0.2 °C was used to measure the temperature. The solidification experiment under identical condition was repeated at least 15 times to calculate the average of the experimental data. The deviations from the mean value were ± 1.5%.

In the analysis of the nanoparticle- and ultrasound-induced water solidification, the states of the cavitation bubbles at different nanoparticle concentrations and ultrasonic intensities were measured using a capillary method [27]. The capillary method involves the attachment of a capillary that can measure the change in volume that occurs due to the formation of large inactive bubbles formed by coalescence among cavitation bubbles. The absorbance values of the aqueous suspension of nanoparticles before and after the solidification/melting cycle were also measured using a UV–vis spectrophotometer (UV9000S, Shanghai Precision & Scientific Instrument Co., Ltd., China) to analyze the dispersion stability of foreign nanoparticles in water during solidification. Five tests were performed for each sample to ensure the reliability of the experimental results.

## Results and Discussion

### Supercooling Degree of Water Controlled by Nanoparticles and Ultrasound Separately

**Fig. 3:**
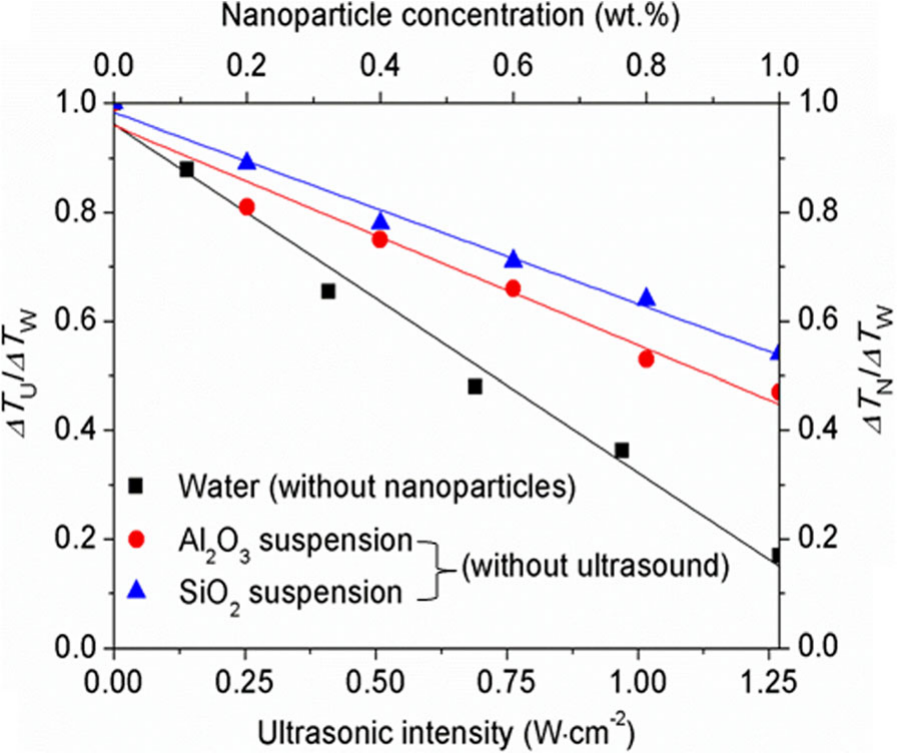
Effects of ultrasound and nanoparticles on the supercooling degree of water. Δ*T*_U_/Δ*T*_W_ represents the ratio of supercooling degree required for the water solidification with ultrasound to that without ultrasound. Δ*T*_N_/Δ*T*_W_ represents the ratio of supercooling degree required for the water solidification with nanoparticles to that without nanoparticles

The ratios of supercooling degree required for the water solidification with nanoparticles to that without nanoparticles (*R*_1_ = Δ*T*_N_/Δ*T*_W_) at different nanoparticle concentrations are shown in Fig. 3. The measured supercooling degree of pure water (Δ*T*_W_) is approximately 11.6 °C. The supercooling degree ratio *R*_1_ is < 1 and decreases with the increase of nanoparticle concentration, which indicates that the Al_2_O_3_ and SiO_2_ nanoparticles can promote the ice nucleation of water as expected. The Al_2_O_3_ nanoparticles have apparently stronger nucleating effect due to the smaller contact angle compared with the SiO_2_ nanoparticles. For example, a 28.3% reduction in the supercooling degree of water is obtained by adding 0.6 wt.% SiO_2_ nanoparticles, whereas at the same concentration, the Al_2_O_3_ nanoparticles can reduce the supercooling degree of water by 37.4%. The weakened nucleating effect of SiO_2_ nanoparticles caused by a big contact angle can be compensated by increasing the nanoparticle concentration. As shown in Fig. 1, a 37.1% reduction of supercooling degree can also be achieved by increasing the concentration of SiO_2_ nanoparticles to 0.8 wt.%. Figure 1 also shows the effect of ultrasound on the supercooling degree of water. The ratio of supercooling degree required for the water solidification with ultrasound to that without ultrasound (*R*_2_ = Δ*T*_U_/Δ*T*_W_) is < 1, suggesting that the cavitation bubbles generated by ultrasound can act as nucleating agents to promote the ice nucleation of water. This nucleating effect of ultrasound can be enhanced by increasing the ultrasonic intensity. In this study, a reduction of 83.1% in the supercooling degree of water can be obtained at the ultrasonic intensity of 1.27 W cm^−2^.

### Supercooling Degree of Water Controlled by Nanoparticles and Ultrasound Mutually

**Fig. 4:**
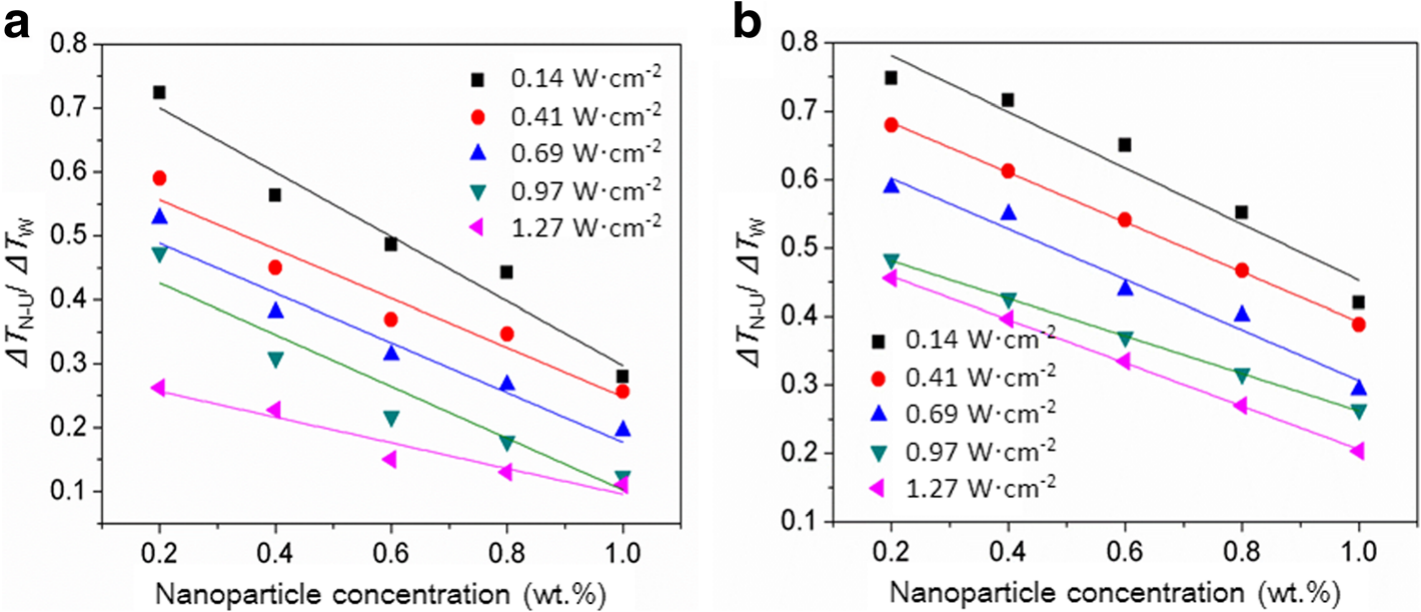
Combined effect of ultrasound and nanoparticles on the supercooling degree of water [**a** Al_2_O_3_ nanoparticles, **b** SiO_2_ nanoparticles]. Δ*T*_N-U_/Δ*T*_W_ represents the ratio of supercooling degree required for the water solidification with nanoparticles and ultrasound to that without nanoparticles and ultrasound

Figure 4 shows the combined effect of nanoparticles and ultrasound on the supercooling degree of water. The ratio of supercooling degree required for the water solidification with nanoparticles and ultrasound to that without nanoparticles and ultrasound (*R*_3_ = Δ*T*_N-U_/Δ*T*_W_) is < 1, indicating that using the nanoparticles and ultrasound mutually can promote the ice nucleation of water in solidification. This nucleating effect of nanoparticles and ultrasound is closely related to the nanoparticle concentration and ultrasonic intensity. For example, a 63.7% reduction in the supercooling degree of water can be obtained at the Al_2_O_3_ nanoparticle concentration of 0.2 wt.% when the ultrasonic intensity increases from 0.14 to 1.27 W cm^−2^. A 58.1% reduction in the supercooling degree of water can be obtained at the ultrasonic intensity of 1.27 W cm^−2^ when the Al_2_O_3_ nanoparticle concentration increases from 0.2 to 1.0 wt.%. The contact angle of nanoparticles is also an important factor that influences the combined effect of ultrasound and nanoparticles. The controlled supercooling degrees of water by Al_2_O_3_ nanoparticles are apparently lower compared with those controlled by SiO_2_ nanoparticles at the same nanoparticle concentration and ultrasonic intensity conditions. For example, the required supercooling degree for water solidification is reduced by 70.6% for the Al_2_O_3_ nanoparticles at the concentration of 0.6 wt.% and ultrasonic intensity of 0.69 W cm^−2^, whereas only a 56.1% reduction of supercooling degree is obtained for the SiO_2_ nanoparticles at the same conditions. To achieve the same 70.6% reduction in the supercooling degree, a higher concentration of 1.0 wt.% is required for the SiO_2_ nanoparticles with large contact angle. Therefore, the ice nucleation of water aided by nanoparticles and ultrasound together can be facilitated by increasing the nanoparticle concentration and ultrasonic intensity and decreasing the contact angle of nanoparticles.

### Comparison of Supercooling Degrees of Water Controlled by Nanoparticles and Ultrasound Separately and Mutually

**Fig. 5:**
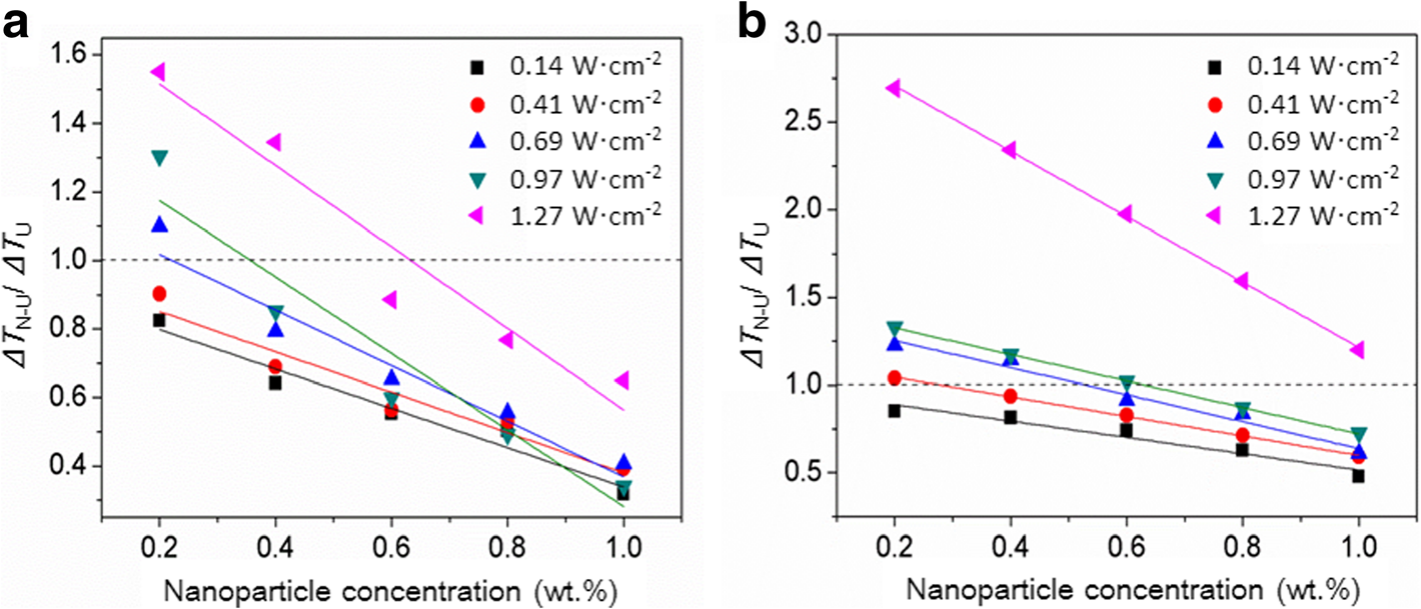
Comparison of the supercooling degrees of water controlled by ultrasound and nanoparticles mutually and separately [**a** Al_2_O_3_ nanoparticles, **b** SiO_2_ nanoparticles]. Δ*T*_N-U_/Δ*T*_U_ represents the ratio of supercooling degree required for the water solidification with nanoparticles and ultrasound to that with ultrasound

When the nanoparticles and ultrasound influence the ice nucleation of water simultaneously, the final effect is found to be not simply the addition of all individual effects; that is, the reduction in the supercooling degree of water determined by the nanoparticles and ultrasound together is actually lower than the sum of the reductions determined by them separately. For example, the supercooling degree of water is reduced by 70.6% at the Al_2_O_3_ nanoparticle concentration of 0.6 wt.% and ultrasonic intensity of 0.69 W cm^−2^ (Fig. 4a), which is smaller than the sum of the 37.4% reduction caused by 0.6 wt.% nanoparticles and the 52.1% reduction caused by 0.69 W cm^−2^ ultrasound (Fig. 3). Furthermore, the reduction in the supercooling degree of water induced by nanoparticles and ultrasound is always larger than that induced by nanoparticles individually, whereas it may be larger than or less than that induced by ultrasound alone, depending on the nanoparticle concentration and ultrasonic intensity. For example, a reduction of 47.2% in the supercooling degree of water is obtained at the nanoparticle concentration of 0.2 wt.% and ultrasonic intensity of 0.69 W cm^−2^ (Fig. 4a), which is larger than the 19.3% reduction caused by 0.2 wt.% Al_2_O_3_ nanoparticles but smaller than the 52.1% reduction caused by 0.69 W cm^−2^ ultrasound (Fig. 3). Figure 5 shows the ratios of supercooling degree for water solidification with nanoparticles and ultrasound mutually to that with ultrasound individually (*R*_4_ = Δ*T*_N-U_/Δ*T*_U_) at different nanoparticle concentrations and ultrasonic intensities. This supercooling degree ratio *R*_4_ of water decreases with the increase of nanoparticle concentration and decrease of ultrasonic intensity; moreover, it is > 1 at low nanoparticle concentrations and high ultrasonic intensities and < 1 at high nanoparticle concentrations and low ultrasonic intensities.

**Fig. 6:**
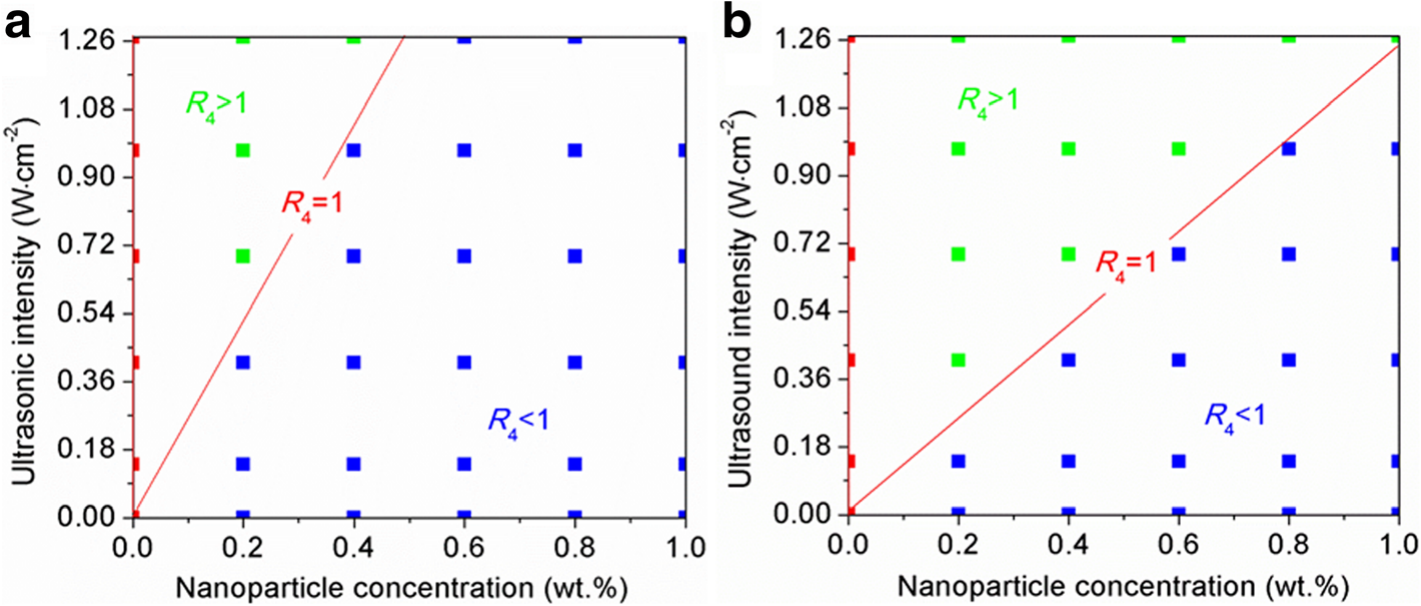
A diagram showing the different effects of ultrasound and nanoparticles on the supercooling degree of water [**a** Al_2_O_3_ nanoparticles, **b** SiO_2_ nanoparticles]. The blue, red, and green dots represent that the required supercooling degree for the water solidification with nanoparticles and ultrasound is lower than, equal to, and higher than that with ultrasound individually, respectively

In this study, we consider that the combined effect of nanoparticles and ultrasound is positive when the supercooling degree ratio *R*_4_ is < 1 and negative when the supercooling degree ratio *R*_4_ is > 1. The corresponding control conditions for these two situations are displayed in Fig. 6. The figure shows a red dividing line on which all the supercooling degree ratios *R*_4_ of water are equal to 1. In the zone above this dividing line (negative zone), all the supercooling degree ratios *R*_4_ are > 1; in the zone below the dividing line (positive zone), all the supercooling degree ratios *R*_4_ are < 1. The nanoparticle concentration and the ultrasonic intensity corresponding to the supercooling degree ratio *R*_4_ of 1 are defined as the critical area and critical intensity, respectively. Apparently, a one-to-one correspondence exists between the nanoparticle concentration and ultrasonic intensity; that is, a higher nanoparticle concentration corresponds to a higher ultrasonic intensity on the dividing line. When the nanoparticle concentration is lower than the critical concentration at a certain ultrasonic intensity or the ultrasonic intensity is higher than the critical intensity at a certain nanoparticle concentration, the supercooling degree ratio *R*_4_ of water will fall into the negative zone, and conversely it will fall into the positive zone. In addition, the critical nanoparticle concentration and ultrasonic intensity are found to be associated with the contact angle of nanoparticles. The comparison of the Al_2_O_3_ and SiO_2_ nanoparticles shows that when the contact angle of nanoparticles increases, the red dividing line of water moves in the direction of high nanoparticle concentration and low ultrasonic intensity, leading to the contraction of the positive zone controlled by nanoparticles and ultrasound together. For example, the controlled supercooling degree ratio *R*_4_ of water by SiO_2_ nanoparticles is located in the negative zone instead of the positive zone at the 0.4 wt.% nanoparticle concentration and 0.69 W cm^−2^ ultrasonic intensity, compared with that controlled by Al_2_O_3_ nanoparticles.

### Nucleation Analysis of Water Solidified Under the Combined Effect of Nanoparticles and Ultrasound

**Fig. 7:**
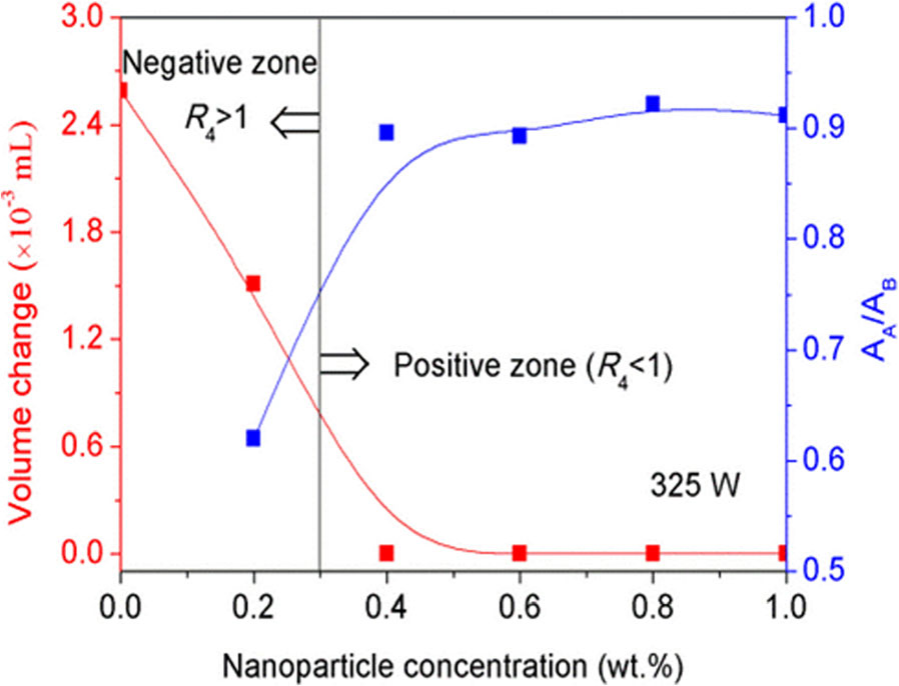
Volume and absorbance variations of the Al_2_O_3_ nanoparticle suspension caused by external ultrasound at different nanoparticle concentrations

**Fig. 8:**
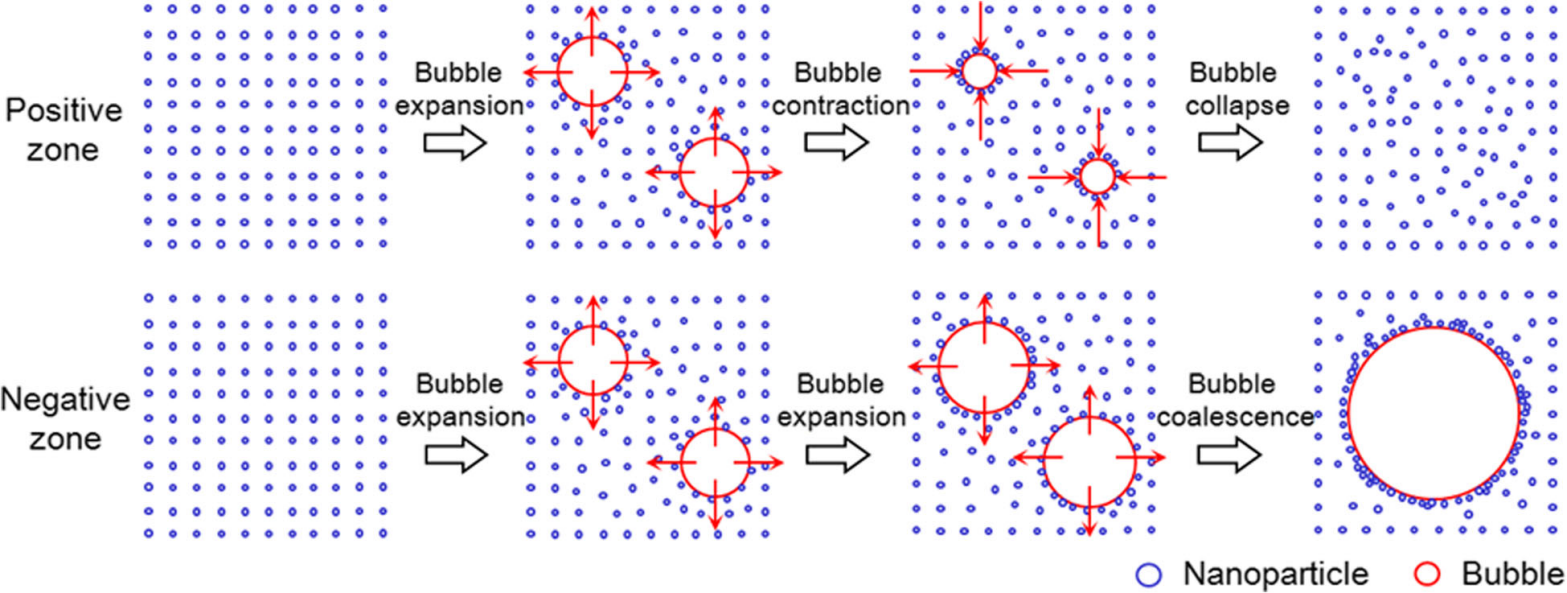
A schematic diagram showing the cavitation bubbles formed in the positive and negative zones

The typical volume changes of water and nanoparticle suspension measured in the positive and negative zones are shown in Fig. 7. In the negative zone, a large volume change is clearly visible, whereas it is completely absent in the positive zone. To the best of our knowledge, two processes, namely rectified diffusion and bubble coalescence, are involved in controlling the growth of cavitation bubbles. Rectified diffusion refers to the growth of a cavitation bubbles due to uneven mass transport across the bubble wall during the rarefaction and compression cycles. During the expansion phase of the bubble (rarefaction), the gases that dissolve in water diffuse into the bubble; meanwhile, during the compression phase of the bubble (collapse), the gases inside the bubble diffuse out of it. Bubble coalescence means that some smaller cavitation bubbles coalesce and form a larger bubble. Unlike the cavitation bubbles formed by rectified diffusion, bubbles formed by bubble coalescence do not undergo the cavitation cycle and do not collapse [28, 29]. We therefore infer that cavitation bubbles in the positive and negative zones may be formed by rectified diffusion and bubble coalescence, respectively. In this study, the dispersion stabilities of nanoparticles during water solidification in the positive and negative zones are also investigated, and the results support the above inference. As shown in Fig. 7, the absorbance ratio (*R*_5_ = *A*_A_/*A*_B_) of the aqueous suspension of Al_2_O_3_ nanoparticles has no considerable change in the positive zone, whereas the absorbance ratio in the negative zone is significantly reduced. The *A*_B_ and *A*_A_ are the absorbance values of the nanoparticle suspension before and after the solidification/melting cycle, respectively. This observation indicates that the dispersion stability of nanoparticles in water can be maintained in the positive zone but deteriorates in the negative zone. In this study, large nanoparticle agglomerates appear in the negative zone, which will settle down quickly in the subsequent melting process. The good dispersion of nanoparticles in the positive zone may be attributed to the impingement of cavitating jets that follow the collapse of gas bubbles formed by rectified diffusion; the aggregation of nanoparticles in the negative zone may be due to the adsorption of nanoparticles onto the large gas bubbles formed by bubble coalescence. The above analysis on the cavitation bubbles formed in the positive and negative zones is depicted in Fig. 8.

Nanoparticles and cavitation bubbles can act as nucleating agents to reduce the supercooling degree of water as stated above. Given that nanoparticles can absorb and scatter the ultrasound energy, the number and size of the bubbles should be decreased. As a result, the nucleating effect of cavitation bubbles possibly weakens in the presence of nanoparticles. Therefore, the superiority of using nanoparticles and ultrasound mutually over using them separately on water solidification depends on whether the nucleating effect of foreign nanoparticles can compensate for the weakened effect of cavitation bubbles. Our experiment results show that in the positive zone (*R*_4_ < 1), the combined effect of nanoparticles and ultrasound is stronger than their respective effects but do not exceed the addition of these respective effects. This result suggests that the weakened effect of cavitation bubbles can be compensated in the positive zone. In the negative zone (*R*_4_ > 1), the cavitation bubbles with large sizes are formed through bubble coalescence pathway, and they have strong adsorption to nanoparticles. Consequently, the combined effect of nanoparticles and bubbles weaken due to the reduction of the total number of the two nucleation sites. This result may be the reason why the required supercooling degree for the nanoparticle- and ultrasound-induced nucleation of water is higher than that induced by ultrasound alone at the same ultrasonic intensity in the negative zone.

Generally, a certain energy barrier has to be overcome to realize the conversion of rectified-diffusion-induced bubbles to bubble-coalescence-induced bubbles. Correspondingly, the rectified-diffusion-induced bubbles can be converted into the bubble-coalescence-induced bubbles by increasing the intensity of ultrasound to some extent. Furthermore, adding nanoparticles with water can favor the formation of rectified-diffusion-induced bubbles by adsorbing and reflecting some energy of ultrasound. In addition, the nanoparticles adjacent to the bubble wall have a shell effect because the cavitation bubbles are generally in the micron range, which is considerably larger than the nanoparticles [30]. The dispersed nanoparticles in water have charged surfaces, and the shell consisting of these nanoparticles should be charged accordingly. As shown in Fig. 1, the nanoparticle suspensions have relatively high zeta potentials. Hence, the coalescence of gas bubbles can be inhibited due to the electrostatic repulsion of the shell, according to the Derjaguin–Landau–Verwey–Overbeek theory [31]. The increase of nanoparticle concentration can certainly strengthen the absorption and shell effects of nanoparticles and thus contributes to the formation of rectified-diffusion-induced bubbles. In short, decreasing the ultrasonic intensity and increasing nanoparticle concentration can facilitate the formation of rectified-diffusion-induced bubbles. Consequently, the critical ultrasonic intensity corresponding to a high nanoparticle concentration, at which the rectified-diffusion-induced bubbles is converted into the bubble-coalescence-induced bubbles, should be higher than that corresponding to a low nanoparticle concentration. Thus, the critical nanoparticle concentration and ultrasonic intensity on the dividing line are positively correlated in this study (Fig. 6). The contact angle of nanoparticles is also proven to be an important factor influencing the ice nucleation of water controlled by nanoparticles and ultrasound. The critical nanoparticle concentration on the dividing line decreases, and the critical ultrasonic intensity increases with the decrease of the contact angle of nanoparticles (Fig. 6). This result may be attributed to the capability of nanoparticles with a small contact angle, which have a strong affinity for water, to be dispersed in the water more steadily and having a stronger shell effect on promoting the formation of rectified-diffusion-induced bubbles, compared with those nanoparticles with a large contact angle.

## Conclusions

In this study, the solidification processes of water under the effects of nanoparticles and ultrasound are investigated mutually and separately. The foreign nanoparticles and cavitation bubbles can act as nucleation sites and promote the heterogeneous nucleation of water. Based on the type of cavitation bubbles generated through ultrasound, we divide water solidification into rectified-diffusion-driven and bubble-coalescence-driven solidification. In the rectified-diffusion-driven water solidification, the foreign nanoparticles can be uniformly dispersed in water exposed to an ultrasound field; thus, the water solidification aided by ultrasound and nanoparticles together can occur at a lower supercooling degree compared with that aided by ultrasound or nanoparticles alone due to the increase in the total number of nucleation sites. On the contrary, the adsorption of cavitation bubbles with large sizes for nanoparticles in the bubble-coalescence-driven water solidification leads to a decrease in the number of effective nucleation sites. As a result, a higher supercooling degree is needed for the water solidification assisted by nanoparticles and ultrasound together. In view of the requirements of low supercooling and high stability for the latent heat storage materials, using ultrasound and nanoparticles mutually is a better method of promoting the ice nucleation of water in the rectified-diffusion-driven solidification compared with using them separately, whereas the situation is reversed in the bubble-coalescence-driven water solidification.

The nanoparticle concentration, contact angle, and ultrasonic intensity are three important factors determining the type of the controlled water solidification by ultrasound and nanoparticles. The critical ultrasonic intensity and nanoparticle concentration, at which the required supercooling degrees for the water solidification assisted by nanoparticles and ultrasound mutually and separately are equal, are found to be positively related and affected by the contact angle of nanoparticles; that is, the critical ultrasonic intensity decreases and the critical nanoparticle concentration increases with the increase of the contact angle. The rectified-diffusion-driven water solidification exists in the zone where the ultrasonic intensity is lower and the nanoparticle concentration is higher than their critical values; otherwise, the bubble-coalescence-driven water solidification exists. Reducing the contact angle of nanoparticles can expand and contract the zones of rectified-diffusion-driven and bubble-coalescence-driven water solidification, respectively.

## Abbreviation

TEM: Transmission electron microscope
